# VETERAN DIRECTED CARE: A MIXED METHODS STUDY TO INFORM SELF DIRECTED CARE PROGRAM EXPANSION FOR VETERANS AFFAIRS (VA)

**DOI:** 10.1093/geroni/igad104.0011

**Published:** 2023-12-21

**Authors:** Stuti Dang, Erin Bouldin, Wendy Hathaway, Pranjal Tyagi, Lauren Penney, Orna Intrator, Samer Nasr

**Affiliations:** University of Miami, Miami, Florida, United States; University of Utah, Salt Lake City, Utah, United States; James A. Haley VA Medical Center, Tampa, Florida, United States; South Florida VA Foundation for Research and Education (SFVAFRE), Miami, Florida, United States; National Institutes of Health, Bethesda, Maryland, United States; Department of Veterans Affairs, Rochester, New York, United States; VISN 8, Miami, Florida, United States

## Abstract

The Department of Veterans Affairs (VA) is shifting from institutional to Home and Community Based Services, such as the Veteran-Directed Care (VDC) program, a self-directed program in which Veterans choose services and employ their providers to avoid or delay long-term institutionalization. The VA plans to expand VDC to all VA facilities by FY2026, but factors affecting VDC expansion are poorly understood. We focused on facilitators and barriers in the seven Veterans Integrated Service Network (VISN) 8 VDC programs between May 2022 and January 2023 to inform program expansion. We used a mixed-methods approach, with surveys followed by semi-structured interviews with VA VDC staff, their partners at Aging and Disability Network Agencies (ADNAs) and VISN 8 leadership, such as lead program coordinators, using the Consolidated Framework for Implementation Research to guide the project rapid analysis techniques. We found substantial variability in the structure, size, and operations of the VDC programs reflecting challenges noted by respondents. VA staff were consistently enthusiastic about VDC’s quality benefits for Veterans, however, they recommended additional staff, a dedicated VDC coordinator, and a national guidebook or standard operating procedures to streamline processes. Respondents also noted the need for more program education targeting referring providers. Unique local challenges, like the lack of available caregivers or no local ADNA, and limited local leadership support were other factors noted that could limit expansion. Importantly, Veterans were often overwhelmed by the employer responsibilities they needed to fulfill. Addressing these concerns remains a challenge to VISN 8 and national leadership.

